# Effect of KDP-Crystal Material Properties on Surface Morphology in Ultra-Precision Fly Cutting

**DOI:** 10.3390/mi11090802

**Published:** 2020-08-25

**Authors:** Dongju Chen, Shupei Li, Jinwei Fan

**Affiliations:** Mechanical Industry Key Laboratory of Heavy Machine Tool Digital Design and Testing, Beijing University of Technology, Beijing 100124, China; zwlshupei@163.com (S.L.); jwfan@bjut.edu.cn (J.F.)

**Keywords:** potassium dihydrogen phosphate (KDP) crystal, material properties, cutting force, surface morphology

## Abstract

To study the effect of material properties on the surface morphology of potassium dihydrogen phosphate (KDP) crystals, an ultra-precision fly cutting machine tool with a single-point diamond tool was used to perform a cutting experiment on (100) crystal plane of the KDP crystal. The elastic modulus, shear modulus, hardness, and dislocation of KDP crystals are taken into the cutting force model by introducing the strain gradient plasticity theory. Since the size effect and dynamic response will affect the surface roughness during ultra-precision machining, the surface roughness of workpieces in ultra-precision fly cutting is hard to predict. Based on the previously established strain gradient plasticity theoretical model, cutting force model, and the dynamic characteristics of the ultra-precision fly cutting system, a surface morphology prediction model under the influence of KDP crystal material properties was established. Finally, the accuracy of the surface morphology prediction model was verified by ultra-precision fly cutting experiments, and identified the frequency range of the characteristic signal caused by the anisotropy of the KDP crystal from the frequency, thereby verifying the KDP crystal material properties has a significant effect on the surface of the machined workpiece roughness.

## 1. Introduction

As an electro-optic crystal material, potassium dihydrogen phosphate (KDP) crystals is widely used in frequency multipliers, three-dimensional optical data storage devices and a new generation of 100 Joule laser systems due to its better nonlinear optical properties [1]. KDP crystals have excellent optical properties as well as special physical material properties such as anisotropy, deliquescent, and easy cracking. KDP crystals are brittle materials on the macro scale, while have plastic deformation ability on the micro scale [2]. The rapid development of solid-state laser technology requires KDP crystals processing surfaces to achieve higher surface quality. Laser damage will caused to the optical components due to the surface topography errors of high-precision optical parts, and thus directly affects its working performance [3].

The chip formation mechanism is different between traditional machining and ultra-precision machining because the cutting depth can reach sub-micron in ultra-precision machining [4]. Traditional machining usually only studies the external conditions such as cutting parameters and tool geometry, while the effect of the material properties is ignored. However, in ultra-precision machining, due to the small processing scale the effect of workpiece material properties on surface morphology cannot be ignored [5]. KDP crystals also has strong anisotropy in mechanical properties same as other single crystals material. A large number of experiments have proved that the Vickers hardness of different crystal faces of KDP crystals is very different, and the magnitude and direction of load have great influence on the fracture behavior of KDP crystals [6,7]. KDP crystal anisotropy will cause the fluctuation of cutting force, which will highly affect surface quality. Zhang et al. [8] explored the microscale removal characteristics of KDP crystals based on a developed ultrasonic vibration-assisted scratch test device. It showed that the material removal characteristics in three directions have significant anisotropy on the (001) plane. Wang et al. [9] studied the cutting mechanism and surface formation process of KDP crystal switches, double-frequency and triple-frequency crystal planes from the basic mechanical properties of materials, and the change law of cutting force and cutting direction was found. Borc J et al. [10] showed the results of the research on the load-displacement curves of (001) and (100) planes of KDP crystals, the impact events and the size effect through the nanoindentation deformation of glass indenters under different loads. In addition, besides KDP crystal, other single crystal materials have been studied by cutting experiments. F. Z. Fang et al. [11] studied the nano cutting mechanism of monocrystalline silicon by molecular dynamics method combined with experiments, and proposed that the chip formation in nano cutting is based on extrusion rather than conventional shear. Y.J.Lee et al. [12] improved the critical cutting depth of CaF_2_ along different cutting directions by coating a solidified coating on the surface of CaF_2_, and the coating did not affect the anisotropy of CaF_2_. Yoon et al. [13] studied the brittle crack and the influence of the secondary cutting direction on the brittle crack during the cutting process of single crystal sapphire. The results show that the defects have a significant impact on the crack propagation in the subsequent cutting process, and the ductile cutting state of sapphire is expanded by about two times by two-step cutting strategy. W.B Lee et al. [14] discovered various deformation characteristics in the process of chip formation by cutting single crystal copper. The results show that the formation of deformation band is direction dependent. However, most of these studies focus on the analysis of experimental results, and there are few theoretical studies on the effect of KDP crystal material properties on surface morphology.

During the process of cutting, the cutting force between the tool and workpiece will cause deformation and vibration both to the tool and workpiece, which will cause the change of relative position of the tool and workpiece, and thus the surface morphology of the workpiece may be changed. C.F Cheung et al. [15] established a surface roughness model considering the effects of tool geometry, process parameters and tool workpiece vibration displacement, which can well predict the surface roughness of diamond turning surface under different cutting conditions. O.E.E.K. Omar et al. [16] mentioned a general and modified model that combines the effects of tool runout, tool deflection, system dynamics, flank wear and tool tilt on surface roughness. Dong et al. [17] developed a generalized kinetic model to study the vibration of the spindle and its effect on surface formation in different UPM processes. Huo et al. [18] established a modified cutting force model and sidewall surface generation model, which considers the effect of tool deflection on cutting force and surface generation. Neto HK et al. [19] explored the influence of cutting strategy, lead angle and tool overhang on cutting force component and workpiece surface roughness, and obtained that surface roughness is related to radial force. Zhou et al. [20] proposed an integrated simulation method involving dynamic cutting process, control system and surface generation. Lu et al. [21] established a flexible deformation model of the micro-milling cutter generated by the cutting force on the basis of previous work. Chen et al. [22] through experimental investigated the effect of anisotropy in ductile cutting of KDP crystals. In addition, when the machining scale is reduced to micron level, the conventional continuum theory is no longer used, so the strain gradient plasticity theory which can better describe the size effect of micro cutting has been developed. Lai et al. [23] used strain gradient plasticity to establish orthogonal cutting model to simulate the strengthening behavior of micro materials, micro edge radius, and fracture behavior of workpiece materials. Giang Na et al. [24] simulated dislocation stacking by choosing appropriate interface conditions and combining with strain gradient plasticity theory. Wang et al. [25] studied the brittle ductile transition mechanism of single crystal silicon (SCS) by using the strain gradient theory. It was found that under certain deformation conditions, not only plastic deformation but also strain gradient effect existed in silicon, which could be attributed to different types of dislocation movement in the crystal. These studies are mainly reflected on the macro scale. For ultra-precision fly cutting, there is lack of more accurate theoretical models to construct the surface morphology.

Obtaining excellent surface morphology is an important index of ultra-precision machining. There are many parameters to characterize the surface morphology, among which 2D surface roughness is the most commonly used. The average line of surface roughness profile is usually determined by least square method as the reference line of contour coordinates [26]. Qu et al. [27] combined the closed form and parametric form into the surface roughness model of $R_{a},R_{q},R_{t}$. Hocheng et al. [28] analyzed the frequency and size of roughness profile, and established the relationship between surface roughness model and tool geometry, low frequency vibration, and measurement accuracy. Bougharriou et al. [29] developed a surface roughness model for *R_t_* that can accurately predict different cutting depths and feed rates. Sun et al. [30] established the surface roughness models of *R_a_* and *R_q_* for wedge angle, cutting edge shape and feed rate. Due to the influence of measuring instrument accuracy and noise error, the traditional measurement parameters cannot reflect the surface characteristics well [31]. Therefore, multi-scale surface information can be obtained by wavelet analysis and power spectral density analysis. Luo et al. [32] analyzed the ultra-precision turning surface by 2D power spectral density method, and found that the local shear stress significantly affected the machined surface morphology, and the feed rate, tool wear and regeneration effect vibration also had some influence. Podsiadlo et al. [33] used fractal and wavelet transform to capture the multi-scale and non-stationary of machined surface and worn surface. Li et al. [34] used wavelet analysis to extract the surface profile, roughness, waviness and flatness of parts, reconstructed the actual surface profile, and generated a virtual surface model with the same contour parameters.

It is of great significance to explore the effect of the material properties of KDP crystals on ultra-precision fly cutting process to improve surface quality. This article starts from the internal structure of KDP crystal materials, comprehensively considers the influence of KDP crystal material properties, establishes a new ultra-precision fly cutting force model, obtains the relationship equation between cutting force and various material parameters, and then establishes a new surface morphology model. By comparing the surface morphology with experimental processing, the influence of the properties of KDP crystals materials on the surface roughness during ultra-precision processing is analyzed. To identify the material properties from the frequency domain, wavelet transform method is used to decompose the detection results of the processed workpiece into signals at various scales, and the power spectrum density method is used to compare the power spectrum of the test results and simulation results. And the frequency range of the characteristic signal caused by the anisotropy of the KDP crystal can be identified from the results.

## 2. KDP Crystal Material Properties Analysis

### 2.1. Mechanical Properties of KDP Crystal

**Figure 1:**
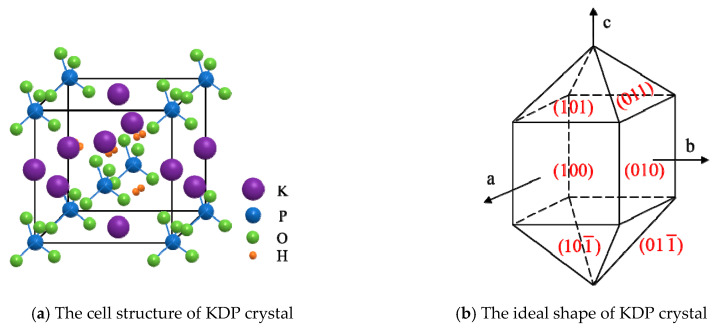
The structure of potassium dihydrogen phosphate (KDP) crystal.

KDP crystals belong to a tetragonal crystal system, with the $D_{2d}-\bar{4}2m$ point group and the $D_{2d}^{12}-I\bar{4}2d$ space group. The cell parameters are a=b=0.7453nm, c=0.6975nm, and Z=4. The cell structure is shown in Figure 1a, and the ideal shape of it is consisted of a quadrangular column and a quadrangular double cone, as shown in Figure 1b.

In the process of ultra-precision fly cutting, the KDP crystal will elastically deform under the action of cutting force. As the increasing of cutting force, yield will occur, and then plastic deformation will occur. In the process of ultra-precision fly cutting of KDP crystals, the shear plane of plastic deformation is the crystal plane with the highest shear deformation specific energy.

**Figure 2:**
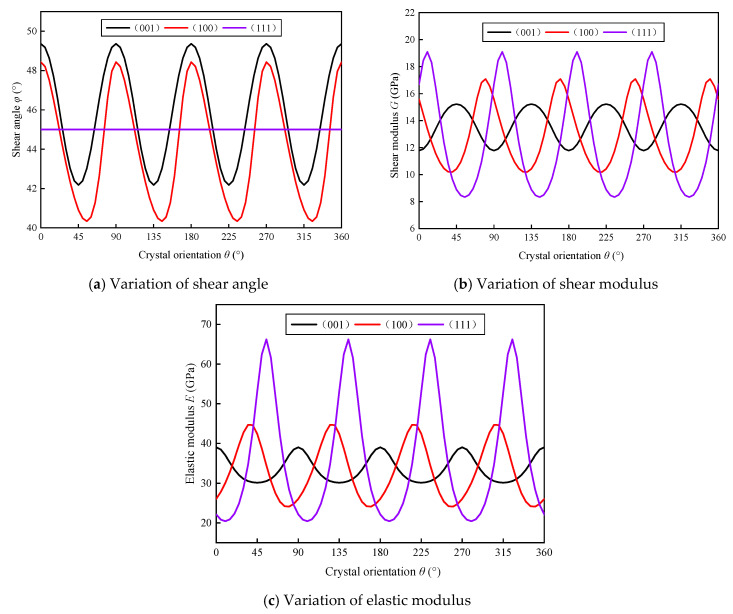
Variation of shear angle, shear modulus, elastic modulus with crystal orientation on different crystal plane of KDP crystals.

By solving the shear deformation specific energy Equation (1), when the equation takes the extreme value, the corresponding ϕ is the shear angle. The shear angle changes of different crystal planes and crystal directions can be obtained by transforming the coordinate system, as shown in Figure 2a. Then, by solving the crystal plane index (*H*, *K*, *L*) and the cosine of the crystal orientation (*R_1_*, *R_2_*, *R_3_*) with respect to the lattice coordinate system, the changes in the elastic modulus E and the shear modulus G of different crystal planes and crystal directions are obtained, as shown in Figure 2b,c. Because the mechanical property parameters corresponding to the (100) crystal plane are relatively small and the variation range is relatively large within one period, the anisotropy is obvious. Therefore, this paper chooses the (100) crystal plane as the research object.

(1) $$c=\frac{F_{s}{}^{2}}{2A^{2}}\cdot \frac{1}{G(\phi )}\cdot {\left[\frac{\sin\phi \cdot \cos(\phi +\beta -\gamma )}{\cos(\beta -\gamma )}\right]}^{2}$$

where *c*, $F_{s}$, ϕ, β, γ, and G(ϕ) represent the shear deformation specific energy, shear angle, friction angle, tool rake angle, and the function of shear angle, respectively.

(2) $$\begin{array}{l} G^{-1}=2S_{11}[R_{1}{}^{2}(1-R_{1}{}^{2})+R_{2}{}^{2}(1-R_{2}{}^{2})]+2S_{33}R_{3}{}^{2}(1-R_{3}{}^{2})-4S_{12}R_{1}{}^{2}R_{2}{}^{2}\\ \;-4S_{13}(R_{1}{}^{2}R_{3}{}^{2}+R_{2}{}^{2}R_{3}{}^{2})+\frac{1}{2}S_{44}(2-R_{1}{}^{2}-4R_{2}{}^{2}R_{3}{}^{2}-R_{2}{}^{2}-4R_{1}{}^{2}R_{3}{}^{2})\\ \;+\frac{1}{2}S_{66}(1-R_{3}{}^{2}-4R_{1}{}^{2}R_{2}{}^{2}) \end{array}$$

(3) $$E^{-1}=(R_{1}{}^{4}+R_{2}{}^{4})S_{11}+S_{33}R_{3}{}^{4}+(R_{1}{}^{2}+R_{2}{}^{2})R_{3}{}^{2}(2S_{13}+S_{44})+R_{1}{}^{2}R_{2}{}^{2}(2S_{12}+S_{66})$$

where *S_ij_* represent flexibility coefficient, its value is shown in Table 1. *R*_1_, *R*_2_*,* and *R*_3_ represents direction cosine.

(4) $$\begin{array}{l} R_{1}=\frac{H}{\sqrt{H^{2}+K^{2}+L^{2}}}\\ R_{2}=\frac{K}{\sqrt{H^{2}+K^{2}+L^{2}}}\\ R_{3}=\frac{L}{\sqrt{H^{2}+K^{2}+L^{2}}} \end{array}$$

where *H*, *K*, *L* represents the crystal plane index, which can be calculated by Equation (5):

(5) $$\left(\begin{array}{l} H\\ K\\ L \end{array}\right)=\frac{1}{\sqrt{3}}\left(\begin{array}{c} \cos\theta \cos\phi -\sin\theta +\cos\theta \sin\phi \\ \sin\theta \cos\phi +\cos\theta +\sin\theta \sin\phi \\ \cos\phi -\sin\phi \end{array}\right)$$

where θ is the angle of crystal orientation.

**Figure 3:**
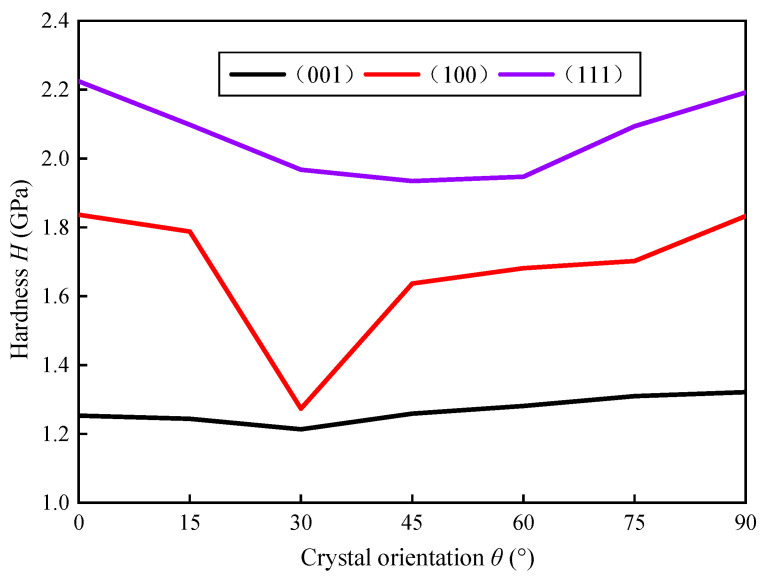
Variation of hardness with crystal orientation on different crystal plane of KDP crystal.

KDP crystals have anisotropic properties, and the mechanical properties of materials in different crystals are different, which will greatly affect cutting performance. In order to study the anisotropy of KDP crystals hardness, it is necessary to perform nanoindentation experiments on KDP crystals. By performing single-point diamond processing on KDP crystals, samples with smooth surfaces with different angles of crystal orientation are processed. Since the mechanical properties of KDP crystals take 90° as a cycle, in the range of 0–90°, with 15° of crystal orientation as an interval, a load of 4000 μN is applied on each crystal surface to perform nanoindentation. By analyzing the nanoindentation experimental data, the hardness value distribution of the different crystal plane of the KDP crystal can be obtained. As shown in Figure 3, the hardness value of KDP crystals in the (100) crystal plane shows obvious anisotropy. The hardness has a minimum value when the crystal orientation is 30°, while the hardness has a maximum value when the crystal orientation is 0°.

It is clearly that KDP crystals have obvious anisotropy by the above analysis. The shear modulus, elastic modulus and material hardness of KDP crystal with different crystalline directions on the (100) crystal plane are all different and change periodically, which has a significant impact on the final processed surface morphology. The anisotropy of KDP crystal is its most important material property, and the anisotropy of the material is of great significance to its processing results.

### 2.2. Establishment of Strain Gradient Plasticity Model

**Figure 4:**
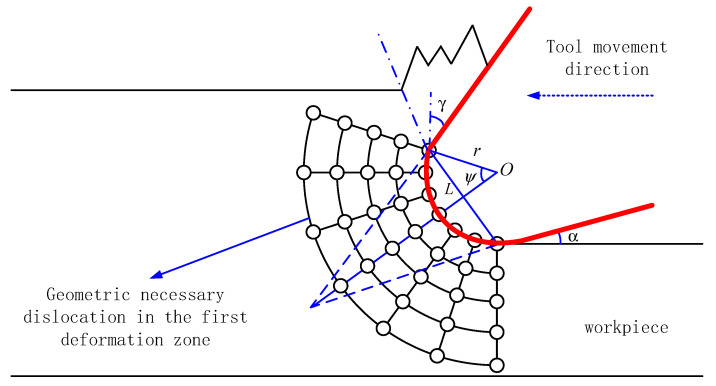
Dislocation mechanism in the first deformation zone of circular arc.

The dislocation mechanism indicates that the plastic hardening of the material originates from statistical storage dislocations and geometrically necessary dislocations. Statistical storage dislocations are related to the plastic strain of the material, and geometrically necessary dislocations are related to the plastic strain gradient of the material. Figure 4 shows the dislocation mechanism in the first deformation zone.

(6) $$\rho_{total}=\rho_{SSD}+\rho_{GND}$$

where $\rho_{SSD}$ and $\rho_{GND}$ represents the statistical storage dislocation density and the geometrically necessary dislocation density respectively.

The relationship between the $\rho_{GND}$ and the plastic strain gradient of the material η can be expressed:

(7) $$\rho_{GND}=l\frac{\eta }{b}$$

where *l* is the Nye factor, which represents the ratio of the $\rho_{GND}$ to the most reasonable dislocation configuration and is generally taken as 3 for single crystal materials, b is the size of the Burger’s vector.

In order to explain the size effect of ultra-precision cutting process, a strain gradient plasticity theory is introduced into the material deformation research during the process of cutting. According to the effective strain gradient model of parallel shear zone [35], the effective strain gradient and geometrically necessary dislocation of the first deformation zone can be expressed:

(8) $$\eta =\frac{1}{L}$$

where *L* represents the length of shear zone.

In the case of chip formation, the thickness of the undeformed chip is larger than the minimum cutting thickness, this is the shear slip phase, and the length of the shear zone is:

(9) $$L=\frac{h}{\sin\left(\phi \right)}$$

where *ϕ*, *h* represents the shear angle and the thickness of the undeformed chip, respectively.

The thickness of undeformed chip can be determined according to the feed rate. Different feed rates can obtain different thicknesses of undeformed chip.

(10) $$f=\frac{V\cdot 1000\cdot h}{\pi \cdot 60\cdot D}$$

where *f*, *V* and *h* represents feed rate, cutting speed, and undeformed cutting thickness, respectively. *D* is the workpiece outer diameter.

(11) $$V=\frac{\pi \cdot D\cdot w}{1000}$$

where *w* is the spindle speed.

(12) $$h=\frac{f\cdot 60}{w}$$

## 3. Establishment of Cutting Force Model

**Figure 5:**
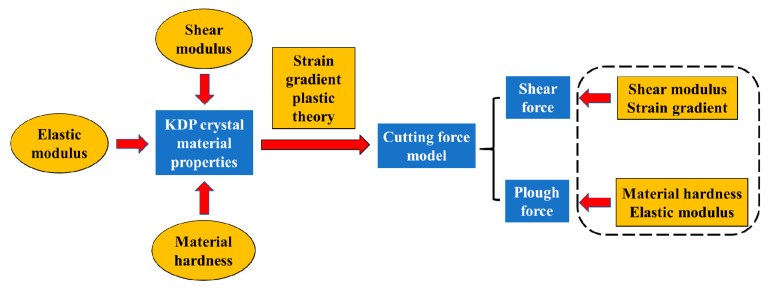
Schematic diagram of the relationship between KDP crystal material properties and cutting force.

During the process of ultra-precision fly cutting of KDP crystals, the mechanical properties of KDP crystal materials will cause the fluctuation of cutting force, which will eventually cause the surface quality of KDP crystals to change. The traditional cutting force model does not consider the influence of material mechanical performance parameters, and often treats some mechanical parameters as constants, making the cutting force model not accurate enough. This chapter starts from the mechanism of ultra-precision machining of the workpiece surface, analyzes the properties of KDP crystal material in the previous section, considers the shear modulus, elastic modulus, and hardness of the KDP crystal, and then combines the theoretical model of strain gradient plasticity to establish newly ultra-precision machining cutting force model. And it can be understood that it is composed of shear force and ploughing force by analyzing the cutting force generated during processing. The schematic diagram as shown in Figure 5.

### 3.1. Shear Force Model

**Figure 6:**
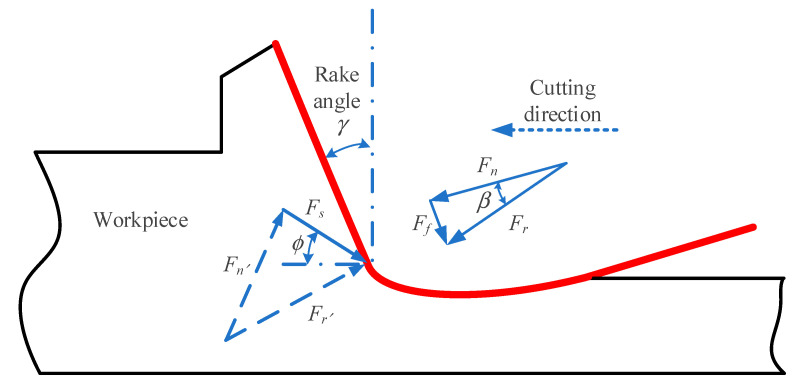
Schematic diagram of shear force in ultra-precision machining.

As shown in Figure 6, the shear force *F_s_* of the shear surface during ultra-precision machining is:

(13) $$F_{s}=\tau \cdot S=\frac{\tau da_{p}}{\sin\phi }$$

where *τ*, *d*, *a_p_*, *S*, and *ϕ* represents the shear flow stress, cutting width, cutting depth, shear surface area, and shear angle, respectively.

As shown in Figure 6, in addition to the shear force on the rake face of the tool, there are positive pressure *F_n_* and friction force *F_f_*. The combined force of the two is resultant force of shear force *F_r_*. In addition, the forces acting on the inside of the material are shear force *F_s_* and normal stress *F_n_*, the combined force of the two is *F_r_*_′_. According to the mutual balance of the acting forces, *F_r_* and *F_r_*_′_ have the same size and opposite directions. According to the geometric relationship, the size of *F_r_* can be determined as:

(14) $$F_{r}=\frac{F_{s}}{\cos(\phi +\beta -\gamma )}=\frac{\tau da_{p}}{\sin\phi \cos(\phi +\beta -\gamma )}$$

where *β* and *γ* represents the friction angle and the tool rake angle respectively. And the direction of the *F_r_* is ρ=β−γ. According to the formulation of Merchant, the *β* can be determined by $\beta =\frac{\pi }{2}+\gamma -2\phi$.

The magnitude of the shear flow stress can be solved based on the effective flow stress, and the relationship between them can be expressed as:

(15) σ=Mτ

where σ is the effective flow stress. According to [36], M is the Taylor factor which acts as an isotropic interpretation of the crystalline anisotropy at the continuum level. *M* is about $\sqrt{3}$ for isotropic materials and about 3.06 for anisotropic materials, so here *M* is set to 3 to simplify the calculation.

(16) $$\tau =\alpha Gb\sqrt{\rho_{total}}$$

where *α* is the material constant coefficient, its value is often 0.3–0.5, *b* is Burger’s vector.

During plastic cutting, due to the ultra-precision machining level, the effective flow stress inside the material under the microstructure needs to be analyzed. A strain gradient is introduced to establish a relationship with the flow stress to describe the size effect in machining process [37].

(17) $$\sigma =M\alpha Gb\sqrt{\rho_{SSD}+\rho_{GND}}=M\alpha Gb\sqrt{\rho_{SSD}}\sqrt{1+\frac{\rho_{GND}}{\rho_{SSD}}}=\sigma_{0}\sqrt{1+(\frac{18\alpha^{2}G^{2}b\eta }{\sigma_{0}{}^{2}})}$$

where $\sigma_{0}$ is the macro model reference stress.

According to the effective flow stress model, it can be found that the size of the shear force is strongly related to the shear modulus *G* and strain gradient η of the KDP crystal.

### 3.2. Ploughing Force Model

In the process of ultra-precision fly cutting, the radius of cutting edge and cutting thickness of the tool are on the same order of magnitude. Under the action of side cutting force, a part of the material becomes the machined surface through extrusion and friction of the cutting edge, and the other part generates plastic lateral flow with the feed of the tool. Therefore, the force acting on the cutting edge of the tool and the flank surface cannot be ignored. When calculating the cutting force, the ploughing force *F_p_* generated by the flank surface should be considered.

(18) $$F_{p}=H\cdot S_{p}$$

where *S_p_* is the contact area and *H* is the material hardness.

**Figure 7:**
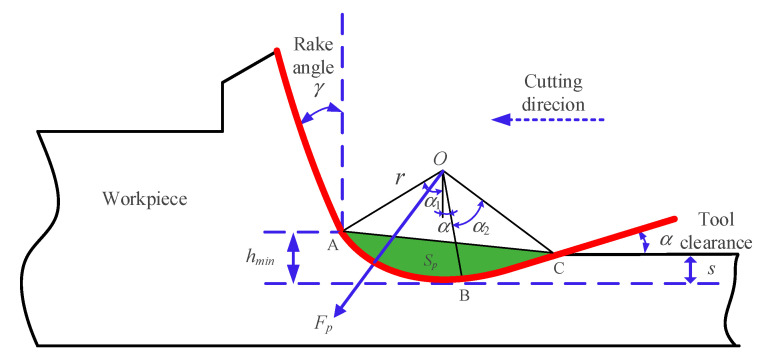
Schematic diagram of ploughing force during ultra-precision machining.

Figure 7 is a schematic diagram of the ploughing force, where the ploughing area is shown in the shaded part of the figure, and the ploughing area can be obtained according to the theoretical formula.

(19) $$S_{p}=S_{AOB}+S_{BOC}-S_{AOC}$$

(20) $$S_{AOB}\approx \frac{1}{2}r^{2}(\alpha_{1}+\alpha )$$

(21) $$\alpha_{1}=\cos^{-1}(\frac{r-h_{\min}}{r})$$

(22) $$S_{BOC}=\frac{1}{2}rl_{BC}$$

(23) $$l_{BC}=\frac{s-r(1-\cos\alpha )}{\sin\alpha }$$

(24) $$S_{AOC}=\frac{1}{2}r\cdot l_{CO}\cdot \sin(\alpha_{1}+\alpha +\alpha_{2})$$

(25) $$l_{CO}=\sqrt{r^{2}+l_{BC}{}^{2}}$$

(26) $$\alpha_{2}=\tan^{-1}(l_{BC}/r)$$

According to the area of the ploughing area obtained, combined with the hardness of the material, the formula for calculating the ploughing force in ultra-precision machining can be obtained:

(27) $$\begin{array}{l} F_{p}=H\cdot S_{p}=H\cdot (S_{AOB}+S_{BOC}-S_{AOC})\\ \;=H\cdot \frac{1}{2}r^{2}\left[\cos^{-1}(\frac{r-h_{\min}}{r})+\alpha \right]+H\cdot \frac{1}{2}r\left[\frac{s-r(1-\cos\alpha )}{\sin\alpha }\right]\\ \;+H\cdot \frac{1}{2}r\cdot \sqrt{r^{2}+{\left[\frac{s-r(1-\cos\alpha )}{\sin\alpha }\right]}^{2}}\cdot \sin\{\cos^{-1}(\frac{r-h_{\min}}{r})+\alpha \\ \;+\tan^{-1}\left[\frac{s-r(1-\cos\alpha )}{\sin\alpha }\right]\} \end{array}$$

where *r*, *s*, and *α* represents the tool nose arc radius, elastic recovery and tool clearance, respectively. μ is the direction of ploughing force, its value is $\mu =\frac{\pi }{4}+\frac{\gamma_{e}+\alpha_{2}}{2}$. $h_{\min}$ is the minimum cutting thickness. According to [38] and our previous work, the minimum cutting thickness of KDP crystal is 0.43–0.48 times of the tool tip arc radius, where 0.43 times of the tool tip radius is taken.

Therefore, the ploughing force of the whole flank $F_{pr}$ can be determined by the following formula:

(28) $$F_{pr}=F_{p}\cdot d$$

where *d* is the cutting width.

When the cutting thickness is very small, the tool can only plough the cutting layer without forming chips. The ploughing action will cause the elastic recovery of workpiece. And the value of elastic recovery can be calculated as follows:

(29) $$s=\frac{3\sigma_{s}}{4E}\cdot r\cdot [2\exp(\frac{H}{\sigma_{s}}-\frac{1}{2})-1]$$

where $\sigma_{s}$ is the tensile strength of workpiece and *H* is the material hardness.

According to the ploughing force model, it can be found that the magnitude of the ploughing force has a great relationship with the material hardness *H* and elastic modulus *E* of the KDP crystal.

### 3.3. Modelling and Simulation of Cutting Force

From the above research, it can be found that during the cutting process, the cutting force is composed of shear force and ploughing force, and its magnitude is closely rely on the material properties of KDP crystals. According to the direction of the shear force and the ploughing force, the magnitude of the main cutting force and the radial cutting force of different crystal orientation in the (100) crystal plane of the KDP crystals can be calculated.

(30) $$F_{c}=F_{r}\cdot \cos\rho +F_{p}{}_{r}\cdot \sin\mu$$

(31) $$F_{t}=F_{r}\cdot \sin\rho +F_{p}{}_{r}\cdot \cos\mu$$

where *Fc*, *Ft*, *F_r_*, *ρ*, and μ represents main cutting force, radial cutting force, resultant force of shear force, angle between the resultant force of shear force and cutting direction, and plough angle, respectively.

**Figure 8:**
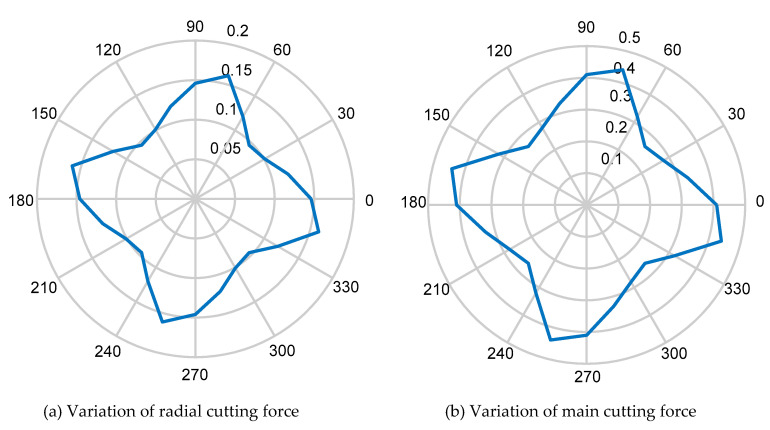
The cutting force with crystal orientation in the (100) crystal plane.

The simulation calculation is carried out by using the commercial mathematics software MATLAB (2016, The MathWorks Inc., Natick, MA, USA) the magnitude of the cutting force of different crystal orientation in the (100) crystal plane can be calculated, as shown in Figure 8a,b. According to [39], the critical cutting depth of KDP crystal in the 45 degree crystal direction is 120–140 nm, and the minimum cutting depth in the 0–45 degree crystal direction is 40–60 nm. Therefore, in order to realize the cutting in the complete ductility region, the cutting depth of 40 nm is selected for the experiment. Table 2 shows the cutting parameters in cutting force simulation.

Comparing Figure 8a,b, it can be found that the main cutting force and the radial cutting force change periodically in (100) crystal plane, and a change period is 0–90°. The magnitude of the cutting force has a minimum value at 45° and a maximum value at 75°. The main cutting force is larger than the radial one.

## 4. Establishment of Dynamic Cutting System and Surface Morphology Simulation Model

**Figure 9:**
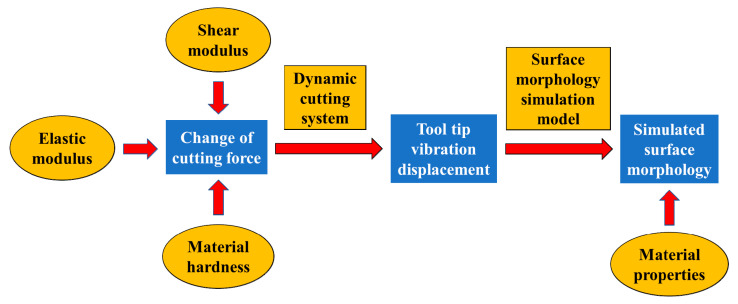
Relationship between cutting force and surface morphology.

According to the cutting force model under the effect of material properties established in the previous section, we can get the cutting force change of different crystal orientation on the (100) crystal plane. And then based on the cutting force model, a dynamic cutting system is established to obtain the displacement of the tool tip with different crystal orientation due to the vibration. Then, the vibration displacement of the tool tip is reflected to the workpiece surface through the surface morphology simulation model, and the simulated surface morphology under the influence of the properties of the KDP crystal material is obtained. The schematic diagram is shown in Figure 9:

### 4.1. Establishment of Dynamic Cutting System

**Figure 10:**
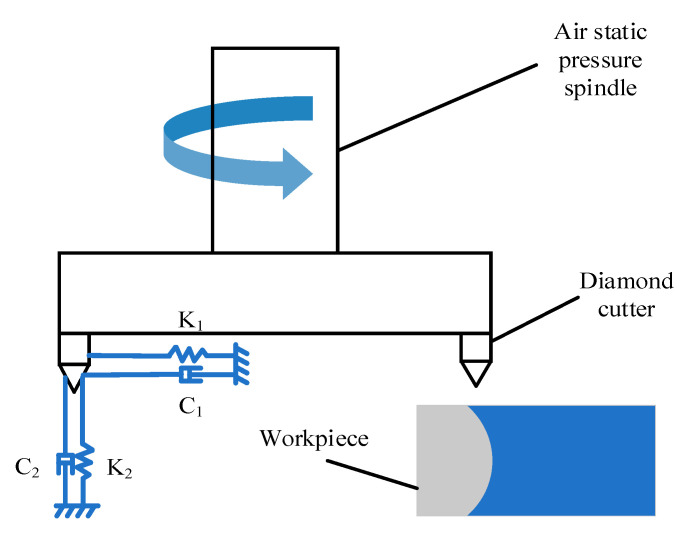
Dynamic cutting system.

In the cutting process, changes in the cutting force will cause tool vibration. During the machining process, the cutting force acting on the tool can be decomposed according to the three directions of the tool axis, feed direction and along the direction of perpendicular to the feed direction. Among them, the biggest component that affect the surface morphology are the force perpendicular to the feed direction and the force along radial direction. Because the feed rate is very small during the process of ultra-precision cutting, the feed force can be ignored. A dynamic cutting system is established according to the ultra-precision machining process, as shown in Figure 10.

Simplify the dynamic cutting system of ultra-precision fly cutting into a two DOF system. By establishing the dynamic differential equation and combining the cutting force model, the corresponding tool tip vibration displacement is calculated.

(32) $$\left\{ \begin{array}{l} M_{1}{\ddot{x}}_{1}+C_{1}{\dot{x}}_{1}+K_{1}x_{1}=F_{c}(t)\\ M_{2}{\ddot{x}}_{2}+C_{2}{\dot{x}}_{2}+K_{2}x_{2}=F_{t}(t) \end{array}\right.$$

Where *M*_1_ and *M*_2_, *C*_1_ and *C*_2_, *K*_1_ and *K*_2_, *x*_1_ and *x*_2_, and *F_c_(t)* and *F_t_(t)* represent mass coefficient, damping coefficient, stiffness coefficient of spindle tool system, vibration displacement of tool, main cutting force, and radial one, respectively.

The modal parameters on the left side of formula (32) are inherent attributes of the machine tool and belong to known parameters. The specific values can be obtained through hammering experiments and modal analysis, as shown in Table 3.

**Figure 11:**
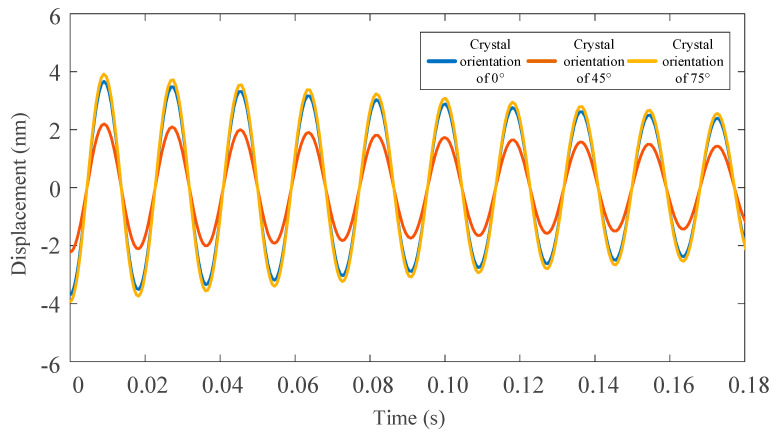
Vibration displacement of three different crystal orientation with tool tip under radial cutting force.

**Figure 12:**
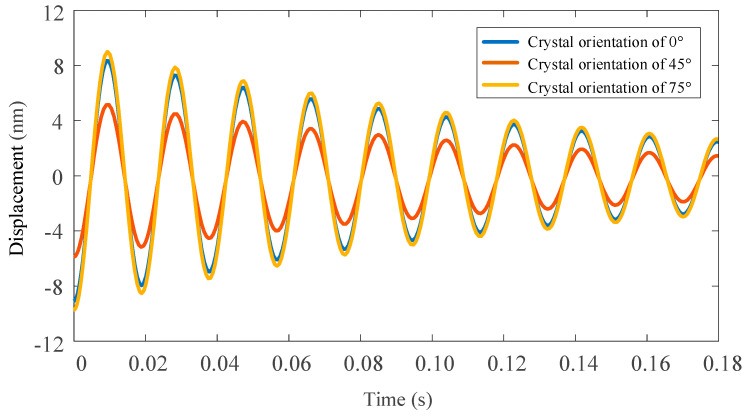
Vibration displacement of three different crystal orientation with tool tip under main cutting force.

The ode function of MATLAB is used to solve the vibration differential equation. Figure 11 and Figure 12 shows the displacement of the tool tip under the action of the main cutting force and radial cutting force by simulating in different crystal orientation of the (100) crystal plane.

In this paper, the least square method is used to fit the vibration displacement curve of the tool tip under the action of the main cutting force and the radial one. Here, the vibrational displacement of the 45° crystal orientation for tool tip under the action of the main cutting force and the radial one are respectively fitted. The fitting equation is shown in the following formula, and the fitting accuracy is 99.2%.

(33) $$\begin{array}{l} X(t)=0.03491-1.407\cos(47.11t)+0.3419\sin(47.11t)\\ \;-1.315\cos(94.22t)+0.6728\sin(94.22t)-1.168\cos(141.33t)\\ \;+1.009\sin(141.33t)-0.9804\cos(188.44t)+1.359\sin(188.44t)\\ \;-0.7983\cos(235.55t)+1.81\sin(235.55t)-0.7556\cos(282.66t)\\ \;+2.937\sin(282.66t)-14.36\cos(329.77t)-0.2538\sin(329.77t)\\ \;-0.6226\cos(376.88t)-1.188\sin(376.88t) \end{array}$$

(34) $$\begin{array}{l} Y(t)=-0.03527-0.2423\cos(46.93t)+0.06268\sin(46.93t)\\ \;-0.226\cos(93.86t)+0.1219\sin(93.86t)-0.1946\cos(140.79t)\\ \;+0.1813\sin(140.79t)-0.1562\cos(187.72t)+0.2414\sin(187.72t)\\ \;-0.1115\cos(234.65t)+0.3187\sin(234.65t)-0.05936\cos(281.58t)\\ \;+0.5071\sin(281.58t)-6.553\cos(328.51t)-0.09671\sin(328.51t)\\ \;-0.09719\cos(375.44t)-0.1988\sin(375.44t) \end{array}$$

### 4.2. Establishment of Surface Morphology Model

Surface morphology modelling is first to determine the three-dimensional trajectory of the tool relative to the workpiece. Among them, the tool tip motion equation is:

(35) $$\begin{array}{l} x(t)=R\cos(2\pi wt)+ft\\ y(t)=R\sin(2\pi wt)\\ z(t)=A\left[1-\cos(2\pi st)\right] \end{array}$$

Where *R*, *f*, *w*, and *s* represents radius of flying cutter disk, feed rate, spindle speed, and relative vibration frequency between tool and workpiece, respectively.

According to the previous content, the vibration displacement **X(*t*) and *Y*(*t*) of tool tip under the action of the cutting force was obtained, and then the vibration equation of the tool tip under the influence of material properties can be obtained:

(36) $$\begin{array}{l} x(t)=R\cos(2\pi wt)+ft\\ y(t)=R\sin(2\pi wt)+X(t)\\ z(t)=A(1-\cos(2\pi st))+Y(t) \end{array}$$

**Figure 13:**
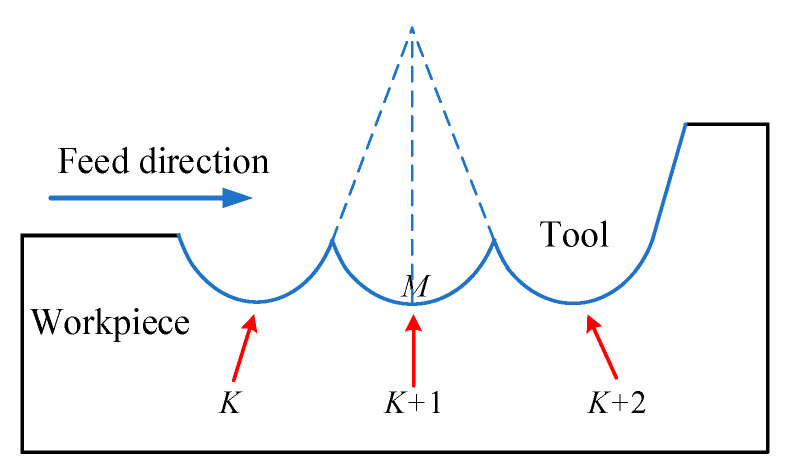
Schematic diagram of tool interference.

Utilizing the law of tool tip movement, the trajectory of tool tip can be regarded as the process of machining the workpiece, and thus calculating the cutting depth corresponding to each node, thereby simulating the 3D machining surface morphology. At the same time, the tool tip arc radius and the interference of the tool are considered. Figure 13 shows that, when the tool and the workpiece are relatively vibrated, the same position will be repeatedly cut. During the simulation process, the maximum value needs to be taken as the actual cutting depth of nodes.

**Figure 14:**
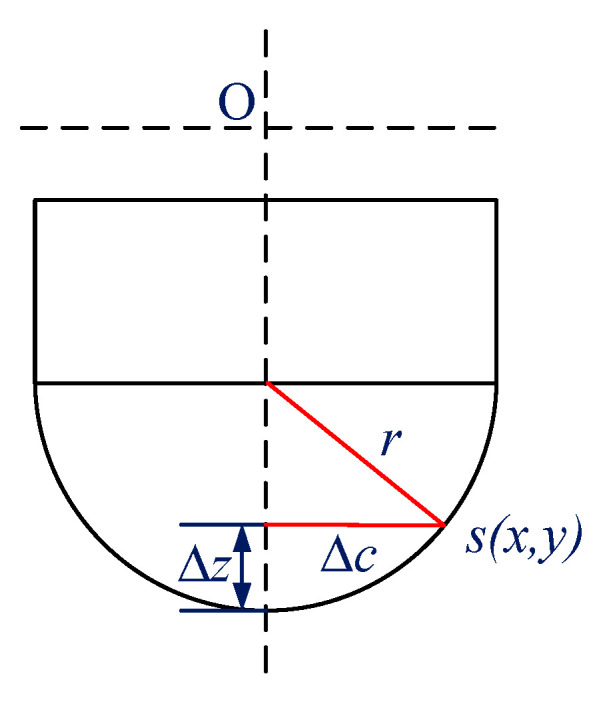
Schematic diagram of arc cutter.

As shown in Figure 14, it is the schematic diagram of arc cutter. The residual height of any point *s*(*x*,*y*) on the workpiece surface can be expressed as:

(37) $$\Delta z=r-\sqrt{r^{2}-\Delta c^{2}}$$

where *r* and Δc represents radius of tool nose arc and horizontal distance between the node and the corresponding tool tip trajectory, respectively.

(38) $$\Delta c=x-\sqrt{r^{2}-y^{2}}+r-f\cdot T(k)$$

(39) $$T(k)=k\cdot dt-\frac{1}{2}dt\cdot \arcsin(y/r)/\pi$$

where *f*, *T*(*k*), and *dt* represents feed rate, processing time of the node relative to the *k*-*th* tool tip movement path, and rotation period of the tool, respectively.

Finally, the cutting depth of the node is calculated, and the cutting depth of the node corresponding to the k-th tool tip movement path is:

(40) z(k)=z(t)+Δz=A[1−cos(2πst)]+Y(t)+Δz

In summary, the actual cutting depth *z*(*k*) of all simulation nodes on the simulation plane can be obtained through theoretical calculation. By mapping the *z*(*k*) values to the coordinates corresponding to the nodes, a three-dimensional simulation image of the surface morphology can be obtained.

By bringing the vibration displacement of tool tip into the surface morphology simulation model, the surface morphology under the influence of material properties can be obtained. Furthermore, the three-dimensional simulation morphology of KDP crystals with different crystal planes and crystal directions is analyzed under the influence of material properties.

**Figure 15:**
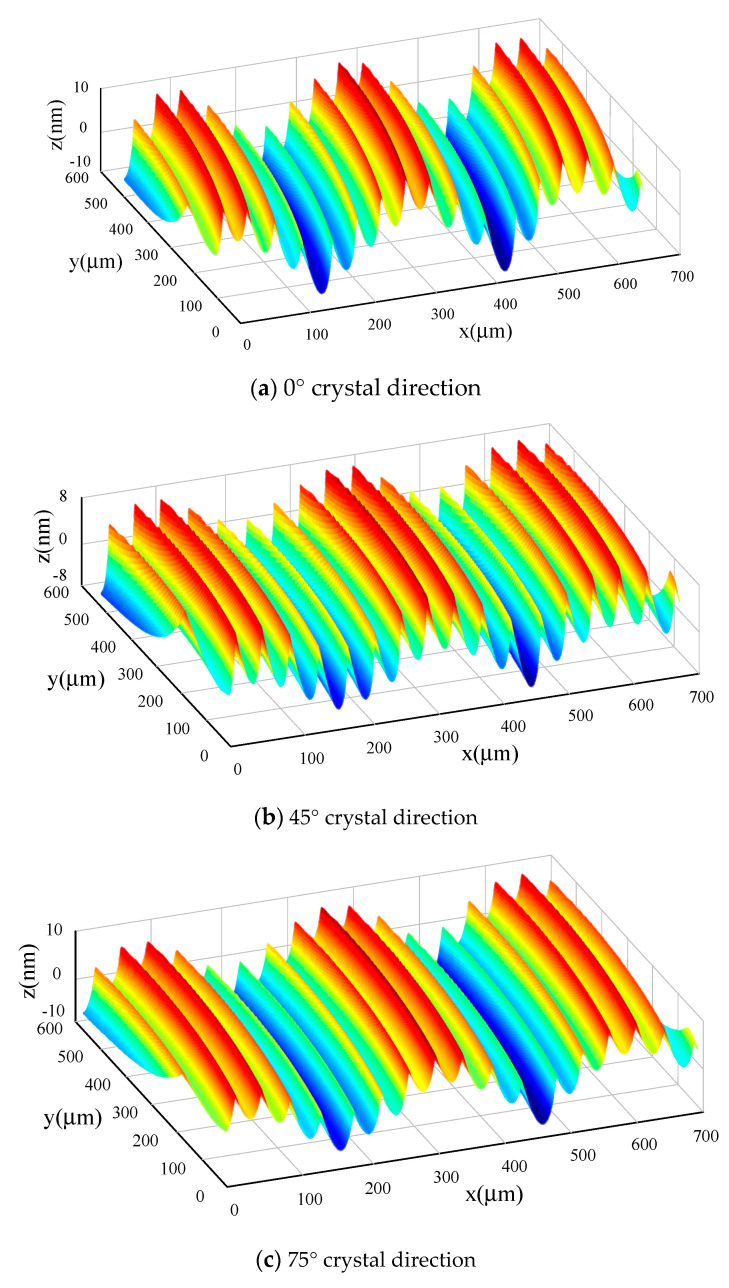
Surface simulation morphology of three different crystal direction on the (100) crystal plane under the main cutting force.

**Figure 16:**
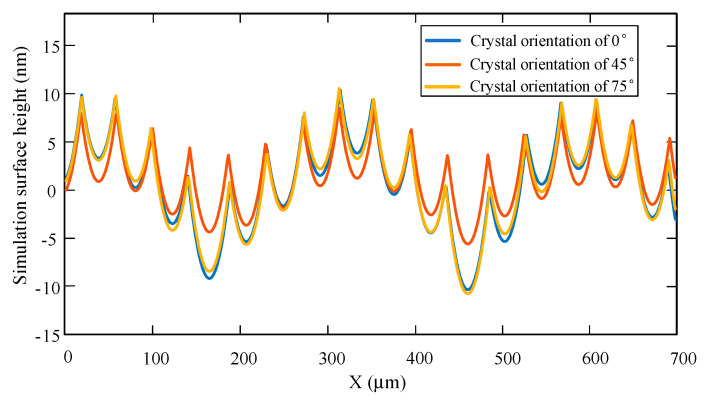
2D surface simulation morphology of three different crystal directions on the (100) crystal plane under the main cutting force.

Figure 15 shows the three-dimensional surface simulation morphology of the (100) crystal plane with three different crystal directions under the main cutting force. The surface simulation range is 700 μm × 525 μm. It can be clearly seen from the simulation images that the surface morphology of different crystal directions on the (100) crystal plane is very different. The fluctuation of the surface morphology on the 45° crystal direction is the smallest, and the surface ripple is low. While the surface morphology on the 75° crystal direction has the largest fluctuation, with a higher surface ripple. The surface morphology changes in the feed direction and perpendicular to the feed direction, and the ripples in the feed direction are more obvious. Therefore, by projecting in the feed direction, Figure 16 shows the simulation morphology of two-dimensional surface with three different crystal directions on the (100) crystal plane under the action of the main cutting force.

## 5. Experimental Results and Analysis

### 5.1. Surface Morphology Analysis

**Figure 17:**
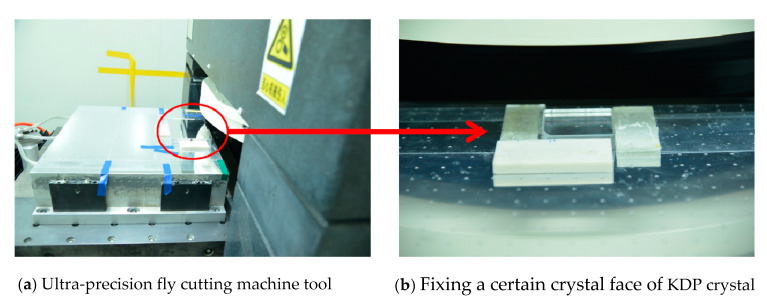
The experimental setup of ultra-precision fly cutting KDP crystal.

Ultra-precision fly-cutting is performed by fixing a certain crystal face of the KDP crystal to obtain a relatively smooth ultra-precision machining surface. Figure 17 shows the experimental setup of ultra-precision fly cutting KDP crystal. This paper sets the cutting depth to 40nm and the tip radius of 10 μm. Table 4 shows the detailed cutting parameters.

**Figure 18:**
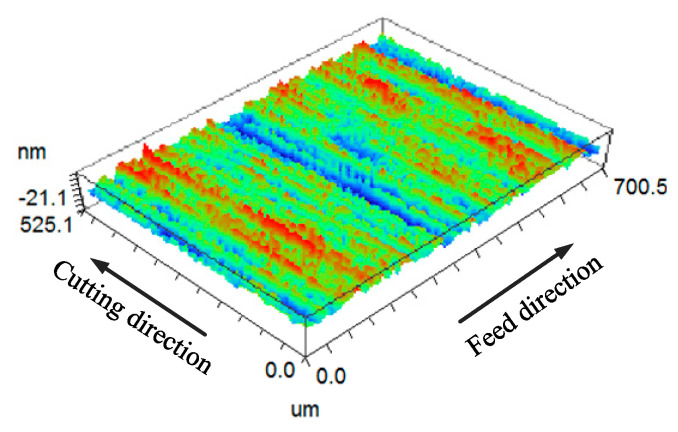
Three dimensional surface morphology of (100) crystal plane.

According to the parameters shown in Table 4, the (100) crystal face of KDP crystal is processed by ultra-precision flying cutting. Figure 18 is a three-dimensional view of the surface morphology of the KDP crystal (100) crystal plane. The surface detection range is 701 μm × 525 μm. It can be seen from the figure that there are obvious ripples existing in feed direction and cutting direction, and in the feed direction, the ripples are more obvious than the cutting direction.

**Figure 19:**
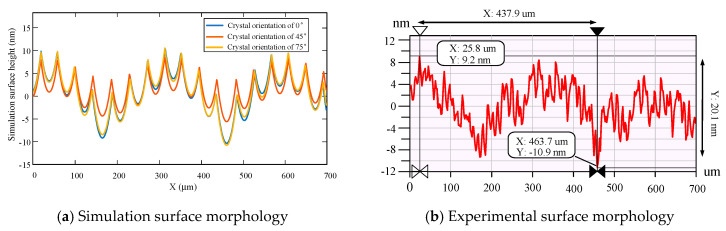
The simulation results and experimental results on (100) crystal plane.

The simulation results and experimental results of KDP crystal are analyzed and calculated. Consider the arithmetic average height *R_a_*, the peak-to-valley height *R_t_*, and the root mean square height *R_q_* of surface roughness. From the perspective of these three parameters, the difference between the two-dimensional feed direction ripple obtained by simulation and the feed direction ripple obtained by experiment is analyzed. The simulation results and experimental results of the (100) crystal plane are shown in Figure 19. It can be seen that the value of *R_t_* is about 20.1 nm, and the generation of the valley in the *x*-distance of 437.9μm may be caused by the tool vibration. By analyzing the ripples in the feed direction, the *R_t_* of the surface roughness of different crystal orientation can be obtained by calculation, as shown in Table 5.

It is necessary to analyze the (100) crystal plane directional characteristics of KDP crystal to explore the directional characteristics of KDP crystal face. The KDP crystal surface takes the interval of 0°~90° with the center of rotation during original cutting as the origin. Measure the surface roughness multiple times every 15° and take the average value. Figure 19 is the surface morphology detection diagram of the observed KDP crystal (100) crystal plane. By analyzing the surface morphology of the (100) crystal plane, the surface roughness values *R_a_* of different crystal orientation are obtained.

Since the roughness measurement index obtained in the simulation is the peak and valley value *R_t_*, and the roughness value obtained in the actual measurement is *R_a_*, it needs to be converted by the following formula:

(41) $$R_{a}=0.2506R_{t}$$

This paper calculates the corresponding surface roughness characteristic parameter *R_q_* (root mean square deviation of surface morphology) for three-dimensional machining surface. For discretely sampled data, the calculation formula is as follows:

(42) $$R_{q}=\sqrt{\frac{1}{l_{x}l_{y}}\int_{0}^{l_{y}}\int_{0}^{l_{x}}z^{2}(x,y)dxdy}=\sqrt{\frac{1}{MN}\sum_{j=1}^{N}\sum_{i=1}^{M}z^{2}(x_{i},y_{j})}$$

where *z*(*x*,*y*), *l_x_* and *l_y_*, *M*, and *N* represents the shape of machining surface, the side length of sampling area, and the number of discrete sampling points in *x* and *y* directions in sampling interval, respectively.

Using WYKO NT9300 optical profiler to detect the surface of the workpiece, the sampling range is 701 μm × 525 μm, a total of 640 × 480 sampling points. Using formula (42) to calculate the *R_q_* of the processed workpiece surface and the simulated surface. Table 5 shows the calculation results.

The roughness prediction error *E* is defined as equal (42):

(43) $$E=\left|\frac{R_{am}-R_{ap}}{R_{am}}\right|\times 100\%$$

where *R_am_* is the actual measurement of surface roughness, *R_ap_* is the simulation value of surface roughness.

Compare the experimental results and simulation results with *R_t_*, *R_a_*, and *R_q_*. Table 5 shows the comparison results.

**Figure 20:**
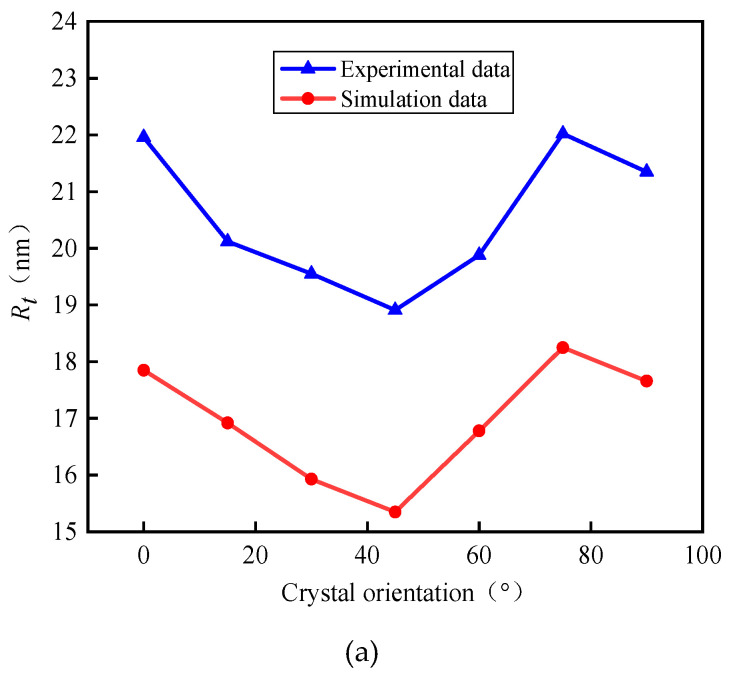

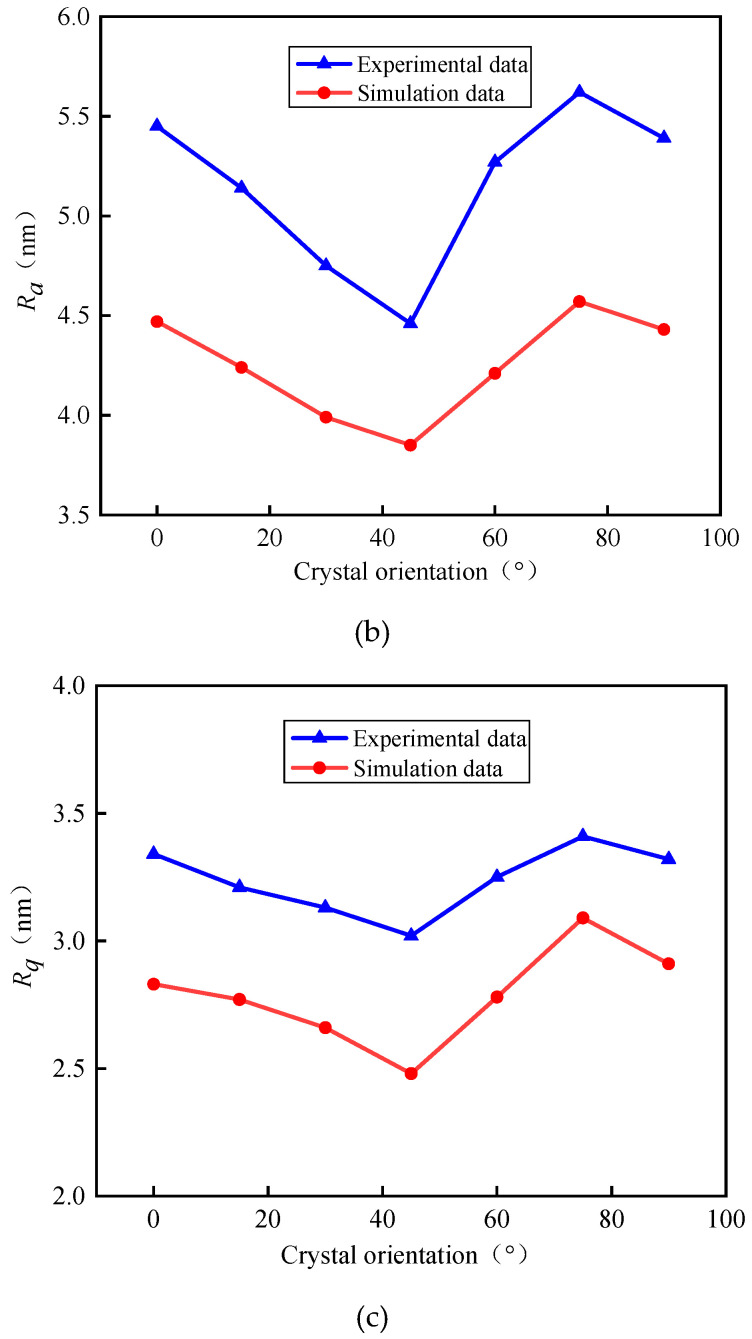
Comparison of simulation results and experimental results. (**a**) Experimental results and simulation results with *R_t_*, (**b**) Experimental results and simulation results with *R_a_*, (**c**) Experimental results and simulation results with *R_q_*.

The surface roughness value obtained in the experiment is compared with the surface roughness value obtained in the simulation, and the comparison result is shown in Figure 20.

Figure 20 shows that both experimental and simulation results have the same trend. In addition, it can be seen from the prediction errors in Table 5 that the error range is between 15% and 20%, which indicates that the simulation model based on the material properties has well verified the change in surface morphology. The results of simulation and experimental show that the surface morphology has obvious anisotropy on the (100) crystal plane, indicating that the surface morphology is affected by the material properties of KDP crystal. However, there is a certain gap between the results of simulation and experimental. The simulated surface roughness is less than the obtained experimental results. The preliminary analysis may be because the vibration of tool and workpiece only considers the impact of material properties. For other factors resulting spindle vibration are not taken into account.

**Figure 21:**
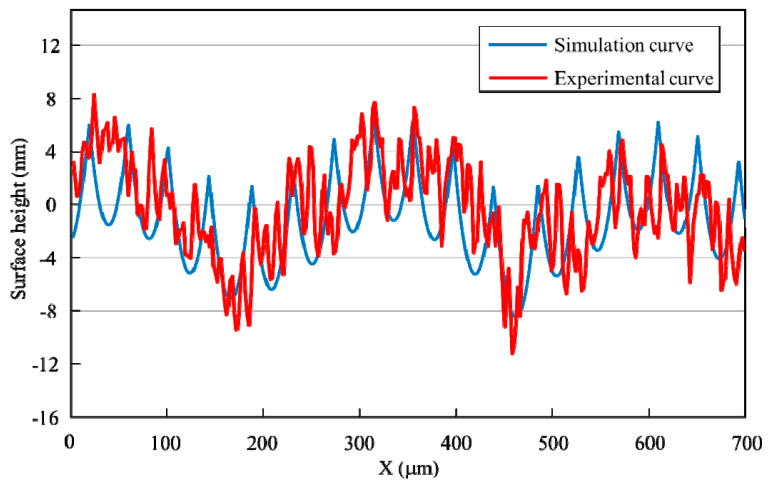
Comparison of simulation curve and experimental curve.

It is necessary to compare whether the ripples of the two results are similar in terms of frequency and amplitude to analyze the waviness of experimental and simulation results. Figure 21 shows a comparison of the cross-sectional profile of the experimental results and the simulation results in the feed direction. From the simulation curve, there are 16 periodic ripples in the length of 700 μm, the frequency is 22.9/mm, and the amplitude is 17 nm. From the experimental curve, it can be seen that there are 19 periodic ripples in the length of 700 μm, the frequency is 27.1/mm, and the amplitude is about 20 nm.

According to the comparison results, the ripple frequency and amplitude of the simulation curve in the feed direction are not much different from those corresponding to the experimental curve. The frequency error is about 15.5%, while the amplitude error is about 15%. The results show that the established simulation model has a certain accuracy and can predict the surface topography well.

### 5.2. Signal Processing and Frequency Identification

The remaining processing traces on the surface of the workpiece will produce noise signals, which makes the collected signals extremely complex. The Power spectral density (PSD) method can separate the specific spatial frequency from the complex signal, and then use the wavelet transform method to multi-scale decomposition of the original surface.

**Figure 22:**
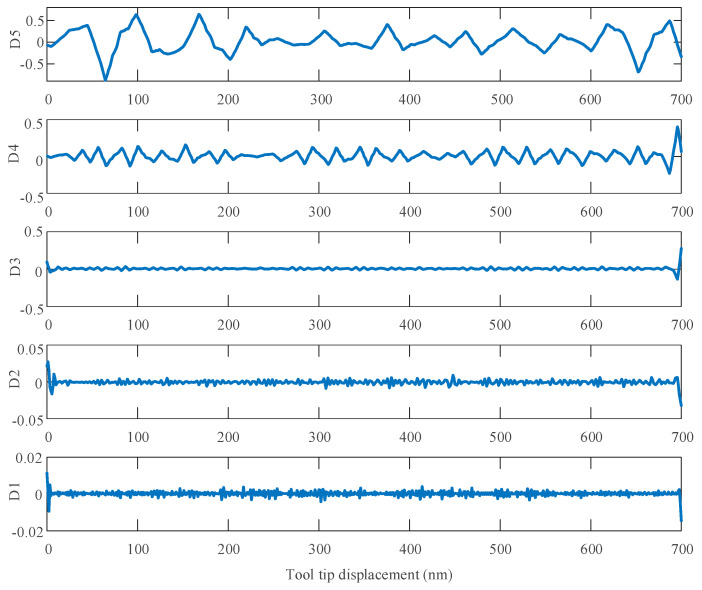
The results of wavelet decomposition.

This paper analyzed the processed surface of KDP crystals by discrete one-dimensional discrete wavelet transform (1D-DWT). Before analysis, the signal should be filtered to exclude the influence of the detection instrument. In this paper, the fifth order decomposition is performed and the frequency information of the (100) crystal plane of the KDP crystal is analyzed by db3 wavelet transform. Figure 22 shows the wavelet transform decomposition results of the detailed signal.

Figure 22 shows the analysis of the power spectral density of the high-frequency information after wavelet decomposition by the Hanning window function. Using formula (44), the description method of the surface contour can be changed from spatial domain to frequency domain, and thus the relationship between surface contour and vibration phenomenon can be identified directly [3]:

(44) $$\omega_{n}=\frac{\pi r\times w\times \cos\theta }{60T_{D}}$$

where *θ*, *T_D_*, and *ω_n_* represents the maximum angle of tangential direction during cutting, the spatial period of surface waviness, and the frequency of surface waviness, respectively.

**Figure 23:**
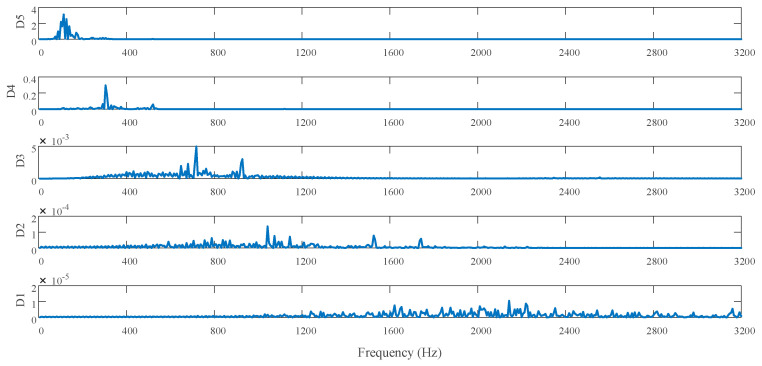
High frequency signal of wavelet decomposition.

According to [40], some ripples in the micro-morphology of the processed surface are related to the physical properties of the material itself. These ripples represent relatively high-frequency information. Therefore, the oscillation frequency caused by KDP crystal anisotropy belongs to the high-frequency signal after wavelet analysis. The scale range of the decomposed signal can be obtained by analyzing the power spectral density of each scale of the high-frequency detailed signal. It can be seen from Figure 23 that D4–D5 has a large surface shape error and its power spectral density is also large, they correspond to the ripples seen on the surface.

**Figure 24:**
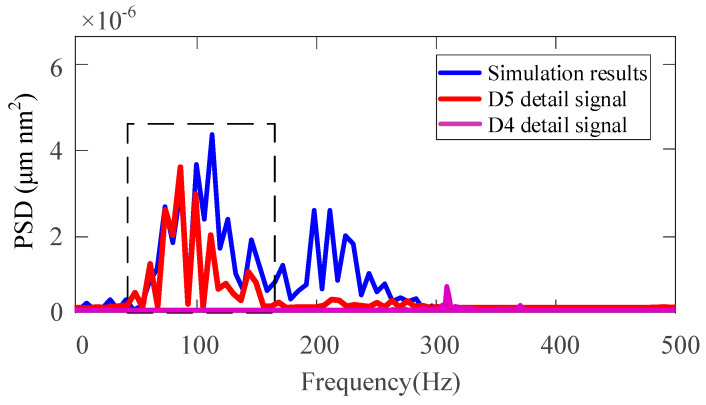
Comparison of power spectrum density between simulation and experimental results.

In the ultra-precision machining process, since the cutting only involves one or a few grains of the material, it is needed to focus on the effect of the material microstructure. The anisotropy of the crystal greatly affects the cutting force and the final surface finish [41]. For the machining surface being studied, the frequency components of D4-D5 are mainly related to the material properties and anisotropic cutting parameters. In order to further obtain the frequency affected by material properties, Figure 24 comparatively analyzes the simulated displacement curve shown in Figure 19a and the power spectral density of the D5 and D4 detail signals. From Figure 24, it can be found that the key signals of the power spectral density of the simulated displacement curve and the power spectral density of the D5 detail signal correspond to each other. In addition, according to the research results in 2.1, it can be found that the anisotropy of the KDP crystal causes its mechanical performance parameters to exist in four periods on one crystal plane, so the frequency caused by its anisotropy should be the same as the rotation frequency of the spindle (*f* = 1000/60 = 16.7 Hz), that is, the frequency should be 66.7 Hz, thus verifying that the frequency range caused by the properties of KDP crystal materials is within the range of 50–150 Hz. The frequency in this range corresponds to a higher power spectrum amplitude, indicating that the material properties of the KDP crystal are the main factors affecting the surface morphology of the workpiece.

## 6. Conclusions

In this paper, the effects of KDP crystal material properties on machined surface in ultra-precision fly cutting are studied using theoretical analysis and experimental verification methods. Based on the material properties of KDP crystals, a flow stress model and a cutting force model are established, and a dynamic cutting model during ultra-precision fly cutting is used to establish a surface morphology model of KDP crystal under the influence of material properties. The effects of the properties of KDP crystals materials on the final processed surface morphology are studied. The main conclusions are as follows:

(1) In order to accurately analyze the material properties of KDP crystals, a shear angle model was first established, and the change of shear modulus and elastic modulus of different crystal planes and different crystal orientation was analyzed. It was found that the shear modulus and elastic modulus change periodically under a specific crystal plane. And by analyzing the hardness and dislocations of KDP crystals, it is found that the hardness of KDP crystals also has anisotropy. In the (100) crystal plane, the hardness decreases first and then increases in the range of 0–90° crystal orientation, and the hardness decreases to a minimum at 30° crystal orientation

(2) A flow stress model and a cutting force model considering the properties of KDP crystal materials are established by introducing the strain gradient plasticity theory. By considering the shear and plough forces in the ultra-precision fly cutting process, the effects of shear modulus, elastic modulus, and hardness of KDP crystal on the cutting force were analyzed. Results show that the cutting force changes periodically, and in the 45° crystal orientation of the (100) crystal plane it is the smallest in one period. Based on this, a dynamic cutting system and surface morphology model were established, and the effect of the vibration response caused by the properties of KDP crystal materials on the surface morphology was analyzed.

(3) Comparing the results of the experimentally processed surface morphology with the simulated surface morphology, it was found that the surface prediction model under the influence of the material properties has a good predictability, and the error is about 15–20%. And it was found that the surface morphology of different crystal orientation has obvious anisotropy. Then use wavelet transform to decompose the surface shape detection result of the (100) crystal plane of the KDP crystal into signals at various scales. Through the power spectrum density analysis, the results of simulation and experimental are compared, and the frequency range of the characteristic signal caused by the anisotropy of the KDP crystal is identified.

## Figures and Tables

**Table 1:** Flexibility coefficient of KDP crystal.

*S*	*S*_11_	*S*_12_	*S*_13_	*S*_33_	*S*_44_	*S*_66_
value(1/100 GPa)	1.51	0.18	−0.40	1.95	7.81	16.2

**Table 2:** Cutting parameters in cutting force simulation.

Machining Parameters	Values
Feed rate f (μm/s)	150
Cutting depth ap (nm)	40
Tool rake angle γ (°)	10
Tool clearance α (°)	5
Radius of tool nose arc r (μm)	10
Cutting width d (μm)	215

**Table 3:** System modal parameters.

	Modal Mass (kg)	Damping	Stiffness (N/m)
X direction	26.8	0.931	18.105
Y direction	27.3	0.381	23.538

**Table 4:** Cutting parameters of ultra-precision fly cutting.

Machining Parameters	Values
Spindle speed *w* (rpm)	1000
Feed rate *f* (μm/s)	150
Cutting depth *a_p_* (nm)	40
Tool rake angle *γ* (°)	−10
Tool clearance *α* (°)	5
Radius of flying cutter disk *R* (mm)	315
Radius of tool nose arc *r* (μm)	10
Workpiece width *W* (mm)	410
Relative vibration amplitude of tool and workpiece *A* (μm)	0.05

**Table 5:** Experimental results and simulation results with *R_t_*, *R_a_*, and *R_q_*_._

Crystal Orientation (°)		0	15	30	45	60	75	90
*R_t_* (nm)	Simulation	17.85	16.92	15.93	15.35	16.78	18.25	17.66
	Experiment	21.96	20.12	19.55	18.91	19.88	22.02	21.35
Prediction error *E*		15.3%	18.7%	15.9%	18.5%	18.8%	15.6%	17.1%
*R_a_* (nm)	Simulation	4.47	4.24	3.99	3.85	4.21	4.57	4.43
	Experiment	5.45	5.14	4.75	4.46	5.27	5.62	5.39
Prediction error *E*		14.3%	18.0%	17.5%	16.0%	13.7%	20.1%	18.7%
*R_q_* (nm)	Simulation	2.83	2.77	2.66	2.48	2.78	3.09	2.91
	Experiment	3.34	3.21	3.13	3.02	3.25	3.41	3.32
Prediction error *E*		14.3%	15.3%	13.7%	15.0%	17.9%	14.5%	9.4%
